# American Medical Association

**Published:** 1854-06

**Authors:** 


### AMERICAN MEDICAL ASSOCIATION.

We proceed to lay before our readers the details of the late meeting of our National Medical Association, at St. Louis, for which we are indebted to the *Republican* newspaper of that city. The attendance of delegates was large, but in casting our eyes over the list, we perceive that the following States were not represented, to wit: Vermont, Delaware, Maryland, North Carolina, Georgia, Arkansas, Florida, Texas, California. Dr. Usher Parsons, of Rhode Island, the Senior Vice President, called the Association to order, when the following letters were read from the President, Dr. Knight.

New Haven, Conn., April 25, 1854.

To Edwin S. Lemoine, M. D., Secretary of the American Medical Association, St. Louis, Missouri.

*Dear Sir:* As the time is near at hand for the assembling of the American Medical Association, it is proper for me to inform you, and through you, the members of the Association, that it will not be in my power to be present at the annual meeting. I have come to this conclusion after much reflection, with great reluctance. The meetings of the Association have always been periods of high gratification to me.

The acquaintance there formed, and the intercourse had with the members of the profession from every part of the country, have been among the most gratifying events of my life. I have looked forward to the annual meeting of this year with the anticipation of unusual satisfaction. There was a portion of the country new to me, to be visited; there were members of the profession, probably in large numbers, few of whom were known to me, to become acquainted with, in addition to the ordinary attractions of the meeting, which made me strongly desirous for my own sake to be present.

At the same time, I feel the full weight of the obligation to attend the meeting this year, arising from the high honor which has been conferred upon me by the Association. Perhaps no one, so little deserving, has received so many and so great favors from the medical profession, and I take the opportunity to renew my acknowledgments for these favors.

It is with no ordinary emotion that, although absent, I greet those of my professional brethren who will be present at St. Louis. I do this with the most cordial feelings, and with the strongest wishes for their welfare. Allow me, also, to express the confident hope that their wise deliberations will result in the promotion of the best interests of the profession, and advantage of the country, and to ask that the blessing of God may rest upon their labors.

With esteem and respect, your obedient servant,

J. KNIGHT.

---

NEW HAVEN, April 15th, 1854.

To USHER PARSONS, M. D., Providence.

*My. Dear Sir:* As the time is near at hand for the meeting of the American Medical Association at St. Louis, it is proper for me to inform you that I shall not probably be able to attend it. Matters of a purely personal nature will, I suppose, deprive me of that pleasure. As you are the Senior Vice President of the Association, I trust you will be present and perform the duties which will devolve upon you. I give you this early notice that you may have time to prepare such an address as may be proper for the occasion. That you will do this as you do everything else, (in the best manner,) I have no doubt.

With esteem and respect, your obedient servant,

J. KNIGHT.

A letter from Dr. BEADLE, one of the Secretaries, stating his inability to attend the meeting, was also read. Dr. WASHINGTON, of St. Louis, then proceeded on behalf of the Committee of Arrangements to welcome the Association to the city. He was followed by Dr. PARSONS in a brief address. The roll having been called, the reports of the Treasurer and the Committee of Publication were read, after which a Nominating Committee was raised to select officers for the ensuing year.

D. ATLEE offered the following resolution which was carried:—That the Association arrange to meet at from 9 o'clock, A. M. to 1 P. M., and from 3 to 5 P. M., while it shall remain in session.

A resolution was now offered to provide for the future meetings of the Association, that they shall be held alternately in the Northern, Southern and Western portions of the Union. The resolution gave rise to considerable discussion, and was finally laid on the table.

On motion the meeting adjourned to 3 o'clock, P. M.

---

AFTERNOON SESSION.

The meeting commenced according to adjournment. The Senior Vice President, in the absence of the President, delivered the following able address:—

*Gentlemen of the American Association:*

It has been customary for the presiding officer of this Association, on retiring from the Chair, to give a valedictory discourse. On the eve of my departure from Rhode Island, our venerable President notified me of his inability to attend and perform this part of his official duty, which deprives us of the rich entertainment anticipated from so distinguished a scholar and professor. The notice being entirely unexpected, I am unprepared to offer you anything worthy of your attention, and my inclination would, therefore, be to remain silent, but for an apprehension that this course might operate as a precedent to others, on similar occasions. I will, therefore, present you, rather as an apology for a discourse, a few thoughts that have suggested themselves while on my way to this city.

In order to promote the honor, dignity and usefulness of our profession, objects for which the Association was instituted, its members must be gathered from all parts of our country and united into one harmonious fraternity, and must adopt such measures as will promote and perpetuate among ourselves an *esprit du corps*, a conformity of sentiment and feeling, and a combination and cooperation in action. This has already been accomplished in a degree by holding our annual meetings in distant and remote cities of the Union. They must continue to be carried to new and ever-varying spheres of action, until their beneficial influence is made available to the whole profession. As the metallurgist, in separating a heterogeneous mass of particles, passes over it a magnetic bar, to attract the pure iron and steel with a force proportioned to its proximity, so must the meetings of this Association, in order to gather into one fold suitable materials of growth and strength, be carried from place to place over the whole mass of our population, attracting from the dross and impurities all that is valuable and worthy of reception and incorporation into a homogeneous and efficient brotherhood. These considerations influenced me in voting to accept the invitation to hold the present meeting in Missouri, notwithstanding the toil and fatigue of the journey, and its remoteness from the residence of a large proportion of the delegates. It is here, more than elsewhere, that the meetings of this

Association are likely to prove beneficial, by a rapid enlargement of our numbers.

Whoever glances at a map of the Mississippi Valley, extending from the base of the Alleghany and Cumberland mountains to the margin of the Rocky mountains—from the highlands bordering on Lake Superior to the Gulf of Mexico—and contemplates the fertility of its soil, its adaptation for cereal productions, which are so necessary for human subsistence and increase, and who surveys the majestic Mississippi, navigable throughout this whole territory with its numerous navigable tributaries pouring in their treasures on either side, and adds to this the vast mineral resources, lead, copper, iron and coal, which are far more conducive to healthful opulence than the golden regions of California; whoever, I say, cordially surveys all these elements of future growth, expansion and power, and moved onward by the agency of steam, on land and water, and in labor-saving mechanical and manufacturing operations, can arrive at no other conclusion than that this vast territory, the largest and most favored one by nature, of any under the whole canopy of Heaven, will in time be densely populated *with scores of millions*, and become the seat of empire of the western world, and that it is destined to be the grand theatre of human progress, in every department that is calculated to advance the dignity and promote the happiness of the human family.

And in no department of human affairs is progress here more sure than in medical knowledge. Our Atlantic States have inherited a reverence for European opinions, which, although commendable in our early medical history, is at the present day, less favorable to American progress and discovery in medicine. We need to interrogate nature and experience more, and European opinions less; we need mental as well as political independence; a freer swing of thought and purpose that characterizes our brethren of the West, and which this Association is adapted to call into action.

There is much to encourage you in your recent discoveries and contributions—in the results of vivisections of saurians, the half of which if confirmed by future experiments, will shed new light on Physiology. And again, in the discoveries made relating to the process of digestion, by your late lamented Beaumont, of St. Louis, who, for the theories and speculations before prevailing, has substituted ocular demonstration of the *modus operandi* of that wonderful process, by submitting to it the various articles of human aliment, and determining the length of time required for converting each into healthful chyme; and again in the successful labors of Drake, in traveling from State to State throughout the valley, collecting the history and character of its epidemics, by personal inquiry and observation. Others of your venerated dead might be mentioned, but of others who have pursued a like independent course untrammeled by prevailing European authorities, who now stand at the head of the profession, it would ill become me to speak, seeing that some of them are present and unused to such freedom of remark. But to the junior members of the profession we would say, unite with us—follow the example of the distinguished pioneers I have named, and of Caldwell and Harrison who have gone to their reward—throw the result of your labors into the common stock of medical knowledge accumulated by this Association, where, rest assured, they will be duly appreciated and be appropriated to the common benefit of the profession and of mankind, and redound eventually to your everlasting honor and professional fame.

Gentlemen, eight years have elapsed since the preliminary meeting of the convention which recommended the formation of this National Association, and the results of its labors have equalled the expectations of the friends of reform and progress in our profession. The six published volumes of transactions have successively increased in value and interest, and are enduring monuments of the ardent zeal and patient industry of the numerous contributors, and there is every reason to hope that our future labors will continue to be crowned with equally increasing value and interest.

Gentlemen, we are reminded by the history of the past year of the frailty and uncertainty of human life. Death has removed many of our brethren of this Association, and among them is the illustrious Beaumont, already mentioned, and Dr. George C. Shattuck, LL. D., of Boston, an extensive and highly esteemed practitioner, and formerly President of the Massachusetts Medical Society.

He was reputed the wealthiest physician in New England, and his numerous bequests to educational, humane and religious institutions and enterprises, and to private charities, proclaim that his philanthropy was proportioned to his opulence.

We are reminded, by the return of this anniversary, of the terrible catastrophe that occurred at Norwalk. The Association had received from our brethren of New York a cordial welcome, and were honored with overflowing hospitality. After a delightful and profitable session, the Association adjourned, and many of them were on their way, in cars, to their respective homes, in joyous anticipation of rejoining their families, when in a moment—in the twinkling of an eye—seven of them were launched into eternity, leaving us the solemn admonition that in the midst of life we are in death. I should deem it a duty, on this occasion, to pay a tribute of respect due to their memory by portraying their many virtues and excellences as men and as physicians, had I not learned that justice will be done them by an abler pen. Our brethren of New York, with characteristic magnanimity, which adds to their claims on our gratitude, immediately on the announcement of the disaster, summoned a meeting, and passed resolutions expressive of their deep sorrow at the sad event, and tendering their condolence to the bereaved families. They also appointed a committee to prepare an eulogy on the deceased, to be offered at this annual meeting; and the distinguished ability of the chairman and members of that committee is a sufficient guaranty that justice will be done to the memory of these, our lamented brethren.

A resolution was now offered that the address be referred to the Committee of Publication for incorporation in the transactions of the Association, which was carried.

The following letter from a permanent member at Marseilles was read and found satisfactory:

Marseilles, March 19, 1854.

Dr. Edward L. Beadle, Secretary of the American Medical Association:

Please to state to the American Medical Association, at their meeting in St. Louis, that I have presented to the Imperial Academy of Medicine at Paris, the sixth volume of their transactions, that it has been received by that distinguished body with much favor, and that it has been referred to a committee for examination and report, (M. Velpeau, Chairman.)

I have also promised M. De Bois, perpetual Secretary of the Academy, to endeavor to procure for the Academy the five previous volumes, and I feel assured that the Academy would take much pleasure in establishing a correspondence with our American Medical Association.

With great respect, yours truly.

The following memorial by members of the Medical Association of the city of New York, was now read:

TO THE AMERICAN MEDICAL ASSOCIATION.

At a special meeting held in the city of New York, on the 12th of May, 1853, of such members "of the American Medical Association as reside in this city and its vicinity, and such as were remaining here from abroad, for the purpose of expressing their feelings respecting the disaster on the New York and New Haven railroad at Norwalk, in Connecticut, which resulted in the death of so many valuable members of the Association," after adopting sundry resolutions expressive of their sentiments and sympathy with the bereaved, a committee of seven was appointed to devise some suitable method of commemorating the event and the worth and professional character of our lamented associates, and to recommend said plan to the next annual meeting of the Association.

At a meeting of the Committee thus appointed it was resolved, that, in the opinion of the Committee, the most appropriate method of carrying into effect the objects had in view in their appointment, would be by preparing a narrative of the event, together with a brief biographical sketch of each individual, which shall embrace a notice of the birth-place, age, place of education, when and where they derived their medical authority, where located after entering the profession, tastes and habits of life, if any, to what particular branch of the profession devoted, what positions held in the profession, either as professors, presidents, or officers of Medical Societies, what literary labors, medical or otherwise, performed, what done to advance the science of Medicine; and that such narrative and biographical memoirs be published in the next volume of the transactions of the Association.

The Chairman and Secretary of the Committee beg leave to state that, although they have taken measures to procure the materials for preparing the Biographical Memoirs, answers to all the letters of inquiry have not been received. In reporting the above proceedings of the Committee to the Association, they would respectfully recommend the adoption of the plan proposed, and suggest that they be authorized to complete the narrative and memoirs in question, and to transmit them to the Committee of Publication.

JOS. M. SMITH, M. D., *Chairman.*

To E. L. Beadle, M. D., *Secretary.*

New York, April 24th, 1854.

The following resolutions, passed at the annual meeting of the New Hampshire Medical Society, were read:

At the Annual Meeting of the New Hampshire Medical Society holden at Concord, June 1, 1853, the following resolutions were unanimously adopted:

*Resolved*, That it is the decided opinion of the New Hampshire State Medical Society that no Delegate should be admitted to membership in the American Medical Association, who represents a Medical Society which numbers among its members any person or persons who adopt as their system of practice any form of empiricism.

*Resolved*, That the Secretary of this Society be instructed to transmit a copy of this resolution to the Secretaries of each of the State Medical Societies, and to the Secretaries of the American Medical Association, previous to their next Annual Meeting.

E. K. WEBSTER,
Secretary N. H. Med. Society.

Boscawen, June, 1853.

Dr. Gross, of Louisville, Kentucky, offered the following resolution:

*Resolved*, That hereafter it shall be considered disorderly for this Association to give costly entertainments.

Some discussion arose, and several amendments were offered; one of which was, that the word improper should be substituted for disorderly, and that liquor and cigars shall be excluded from such entertainments.

Dr. Coons, of St. Louis, remarked in this connection, that the objects of the Society had been more directed to efforts of entertainment than to promote science. Several members advocated the adoption of the resolution without reference to what had taken place, but to provide against the practice becoming an evil in future. The resolution finally passed as amended.

The Committee for the nomination of officers now appeared, and made the following report:

*President.*—Charles A. Pope, M. D., of Missouri.

*Vice Presidents.*—E. D. Fenner, M. D., of Louisiana; N. S. Davis, M. D., of Illinois; Wm. T. Wragg, M. D., of South Carolina; John Green, M. D., of Massachusetts.

*Secretaries.*—E. S. Lemoine, M. D., of Missouri; Frank West, M. D., of Pennsylvania.

*Treasurer.*—D. F. Condie, M. D., of Pennsylvania.

The report was accepted, and the officers called to fill their several stations. Dr. Storer, of Boston, Dr. White, of Buffalo, Dr. Brainard, of Chicago, and Dr. Reed, of Tennessee, were appointed a committee to conduct the newly appointed officers to their seats on the platform.

Dr. Pope was not in attendance on account of sickness in his family, and the Senior Vice President was called upon to officiate in his stead. Dr. Fenner returned thanks for the honor conferred upon him, and regretted the absence of the President.

On motion, the city of Philadelphia was fixed upon as the next place of meeting of the Association.

Dr. Condie, Chairman of the Committee of Publication, offered certain resolutions to the effect, that yearly dues of the members of the Association should not be less than three dollars, and insisting on their being regularly paid. The resolutions, after considerable discussion, were adopted.

A resolution adopted by the Detroit Medical Association, extending an invitation to the Association to hold its next annual meeting in Detroit, was now read. It was received and laid on the table.

Dr. Atlee, on behalf of the committee to procure a stone with a suitable inscription for the monument of Washington, reported that he had adopted, at the suggestion of the lamented Dr. PIERSON, of Salem, the design for the stone, representing HIPPOCRATES refusing the presents of King ARTAXERXES, who invited him to go to Persia and succour the enemies of Greece. The sculpture was on beautiful marble, by SAMUEL BECK, a young artist of Lancaster county, Pennsylvania, from a daguerreotype copy of VIARDOT's celebrated picture, presented to him by Miss ABBY L. PIERSON. The execution of the work is in the highest style of art, and evinces extraordinary talents in the artist.

The stone is of Vermont marble. The resolution authorizing the movement was adopted at Richmond. There was a lack of funds for the accomplishment of this object to the amount of four hundred dollars, and members of the Association were respectfully invited to contribute, as they felt inclined, to make up the amount.

On motion, Dr. CHARLES HOOKER was appointed Treasurer pro. tem., as the Treasurer elect was not in attendance.

A gentleman now announced to the meeting that Dr. POPE, the President elect, was in the room. Two of the committee appointed for that purpose then escorted the President to the chair. He addressed the Association in a few remarks as follows:—

GENTLEMEN: There are occasions when the mouth is dumb, because the heart is full. Such I feel my present position when I behold around me so many members of a noble profession. I am grateful for the honor you have conferred upon me, and however unworthy in other respects, I will yield to none in a just appreciation of the lofty and noble profession of which we are members. In this view, Gentlemen, I feel that the honor was not so much intended for myself as for the advances made in science by the profession generally, in the West. For myself, I return you the thanks of a grateful heart. I will endeavor to act to the best of my abilities, and again I thank you.

Dr. NINIAN PINKNEY, of the U. S. Navy, addressed the Association on the re-organization of the Medical Bureau of that branch of the public service.

The address was listened to with marked attention, and the eloquent style and impassioned delivery of the speaker, proved him at once an enthusiastic lover of his profession, and possessed of a mind capable of mastering its subtleties and advancing its interests in every department. His address explains itself, and will, we doubt not, prove interesting to members of the faculty, as well as to the general reader.

A communication was received from the Hon. L. M. KENNETT, inviting all the members of the delegation and the Faculty of the city in general, to partake of the hospitalities of his house, in an entertainment to be given in the evening.

A communication was also read from the President of the Board having charge of the Institution of the Blind, of St. Louis, inviting the members of the delegation to visit that Institution during their stay in the city.

A resolution was adopted authorizing the Committee of Nomination to nominate and appoint other committees that may be needed during the session.

Drs. MOORE, MCPHEETERS and REYBURN, extended general invitations to the members of the delegation to visit them at their residences.

The meeting then adjourned to nine o'clock next morning.

---

### SECOND DAY—WEDNESDAY.

The meeting convened as per adjournment at nine o'clock, A. M., Dr. POPE, President, in the Chair.

The minutes of the preceding day were read, and after one or two slight amendments, were adopted.

On motion, it was resolved, that the Association adjourn the afternoon session at 4 o'clock, P. M.

Dr. ATLEE, of Lancaster, Pennsylvania, moved that the memorial from the members of the American Medical Society of Paris to the American Medical Association, be read and referred to the Committee of Medical Education, which motion carried. The memorial is as follows:

*Memorial of the American Medical Society of Paris, to the American Medical Association:*

We, the members of the American Medical Society of Paris, beg, through our delegates to present the following memorial:

The National Association of the United States has had its origin mainly from the consciousness felt by physicians of the low state of medical education in our country, and from the desire universally entertained by them of elevating the standard of the medical profession. We, by our sojourn abroad, from our intercourse with those educated here, have become more painfully conscious of our infirmities and deficiencies at home, and for this reason beg leave once more to urge upon the Association the necessity of a change. While acknowledging, however, the superiority of education in Europe, we are far from desiring to arrive at equality by imitating their methods. We therefore beg to urge the following plan for the consideration of the Association: That in each State there be appointed by the Medical Society of the State a Board of Examiners, which Board shall be chosen every year from members of the Society, and which shall perform its duties the following year in the place, and immediately before the sitting of the State Medical Society; that their examinations be public, and that every one whomsoever may apply who shall be introduced by a member of the Society; and that no one can hereafter become a member of the State Medical Societies nor of the American Medical Association who has not the certificate of having satisfactorily passed such an examination. As to the qualifications to be required of the candidate, we do not think it advisable to enter into particulars. They should not, however, believe in any peculiar doctrines or methods; no certificates of attendance upon courses of lectures should be necessary, but solely the possession of the necessary amount of medical knowledge to practice his profession with safety and honor. This plan in no way interferes with the established schools; its effect upon them could only be salutary. Students would attend those institutions where those branches of a medical education that can only be acquired by attendance upon lectures are best taught.

All of which is respectfully submitted to the consideration of the Association.

Dr. HAMMER, *St. Louis.*
Dr. MURPHY, *Cincinnati.*

The President announced the reading and consideration of the annual reports of committees would now be in order. The follow ing chairmen of committees submitted abstracts of their reports or the diseases and epidemics committed to their investigation, as appearing in their several sections and districts.

Dr. F. Condie, of Philadelphia, on the Causes of Tubercular Disease, was not prepared to report, and requested further time.

Dr. Geo. B. Wood, of Philadelphia, on Diseases of Parasitic origin not being present, had sent a verbal request to be discontinued. His request was accordingly granted.

Dr. John A. Atlee, of Lancaster, Pa., on Epidemics of New Jersey, Pennsylvania, Delaware, and Maryland, not being prepared to make a full report, requested to be continued on the same committee.

Dr. D. J. Cain, of Charleston, S. C., on Epidemics of South Carolina, Florida, Georgia and Alabama, read an abstract of his report. It was referred to the Committee on Publication.

Dr. W. L. Sutton, of Georgetown, Ky., on Epidemics of Tennessee and Kentucky. He had made a partial report, but of such meagre materials that he requested to be continued. His report was referred to the Committee of Publication when ready.

Dr. George Mendenhall, of Cincinnati, O., on the Epidemics of Ohio, Indiana, and Michigan. He presented a report for the years 1852 and 1853, from which he read a brief abstract. The report was referred to the Committee on Publication with the request to have it published in the proceedings of the present year.

Drs. PALMER and ATLEE each spoke at some length of the great and lasting good that might be accomplished if all the members of the Association would duly record their individual experience of epidemics, and report all such cases to the Chairmen of Committees. Dr. ATLEE had been two years Chairman of such a committee and during that time had only received two such reports, one from New York, and a partial one from Pennsylvania. He stated that he had used great exertions to get professional men to co-operate in this work, and appealed to the whole faculty to do everything in their power to promote this great object. He very ably impressed the necessity of co-operation by all the profession with the several committees having these reports in charge.

Dr. R. S. HOLMES, of St. Louis, Mo., on Epidemic Erysipelas read an abstract of his report. It was referred to the Committee on Publication.

Dr. E. D FENNER, of New Orleans, on Epidemics of Louisiana, Mississippi, Texas and Arkansas. He read a comprehensive abstract of his report—dwelling principally on the ravages of the cholera and yellow fever, and the causes and the means of treatment.

Dr. FENNER had not completed his report, and Dr. McPHEETERS offered a resolution that Dr. FENNER be requested to complete his report, and submit it to the Committee on Publication to be published. The resolution was adopted.

Dr. MUSSEY, of Cincinnati, now made a motion to suspend the order of regular business, to allow Dr. LINTON, of St. Louis, to express his views with regard to the Pathology of the Yellow Fever. A suspension of business was made for this purpose.

He expressed his views very clearly and at some length on this subject. He advocated the idea that vegetable decomposition was not necessary to the production of the autumnal diseases of this country. He considered yellow fever nothing more than an aggravated type of bilious fever, caused by the retention of hydro-carbonaceous substances in the blood. In other words, the agencies producing yellow fever where *Northern blood* subject to the heat of Southern latitudes.

A motion was made and carried, that Dr. LINTON be requested to draw up the substance of his remarks, to be presented to the Committee of Publication.

Dr. DANIEL BRAINARD, of Chicago, Ill., on the Constitution and Local Treatment of Carcinoma. He requested further time to make a full report.

Dr. N. S. Davis, of Chicago, Ill., on the Influence of Local Circumstances on the Origin and Prevalence of Typhoid Fever. The report, of which he read a brief abstract, was referred to the Committee on Publication.

Dr. Donaldson, of Baltimore, on the Present and Prospective value of the Microscope in Disease. Dr. Donaldson, in a communication, stated that his report was complete, but he not being present, it was, without reading, referred to the Committee on Publication.

The report of the Committee on Medical Education was received but owing to its length, its reading was passed over. It was referred to the Committee on Publication.

Dr. Pope, Chairman of the Committee on Prize Essays and Volunteer Communications, now made the following report:

Mr. President: The Committee on Prize Essays and Volunteer Communications, respectfully report that the Essays submitted to their consideration were nine in number, of which one was presented as a volunteer communication. The committee have carefully examined the whole of these Essays and bestowed upon them the attention which a sense of the importance of the duty assigned them imposes. They feel free to say that some of these Essays possess undoubted merit, both in matter and style, and they admit in them evidence of high scientific attainment as well as a familiarity with the graces of composition. But whilst cheerfully according these claims to their authors, the committee have preferred to be governed in their choice by considerations of originality and practical import, rather than of mere theoretic speculation, however finely portrayed. The committee, have, consequently, concluded to award but a single prize. The Essay selected is entitled "An Essay on a new method of treating Ununited Fractures and certain Deformities of the Osseous System." It bears a motto in French, which, being literally rendered in modern English, reads, "and notwithstanding all the pains I have heretofore taken, I have reason to praise God, in that it hath pleased Him to call me to that branch of Medical practice commonly called Surgery, which can neither be bought by gold nor by silver, but by industry alone and long experience."

If it please the Association I will now break the seal of the packet superscribed by the same motto, and declare the name of the successful competitor.

Dr. Pope then broke the seal and announced the name of Prof. Brainard of Chicago.

Dr. McPheeters moved that Prof. Daniel Brainard take the stand and give the Association an abstract of his new mode of treating fractures, &c., which motion was carried, and Prof.

BRAINARD accordingly came forward, and in an able manner gave the requisite information.

Dr. HOOKER, of Connecticut, Treasurer, now introduced the subject of annual assessments, and called the attention of the Association to the fact that he was ready to receive the dues of members.

Dr. ELBERT offered certain resolutions to the effect that a committee be appointed to recommend to the next annual meeting, for consideration, any alteration they might deem necessary in the Constitution, By-Laws, &c., and also that the place of holding future annual meetings of the Association be determined by ballot, without the intervention of the Nominating Committee. The resolutions, after much discussion, were lost.

Dr. GUTHRIE offered the following resolutions, which were unanimously carried:

*Resolved*, That in the Secretary of the Treasury's recommendation to Congress to abolish or materially modify the duty on such crude drugs not producible in this country, as are used in the laboratories of the country in the manufacture of chemicals, we recognize a wise provision for the future protection of the profession and the community at large, from impure and sophisticated medicines.

*Resolved*, That a copy of this resolution be signed by the proper officers of this Association, and transmit the same to the Secretary of the Treasury and to the Committee on Ways and Means.

Dr. J. B. JOHNSON now stated to the meeting that he had a letter from Dr. STEPHEN WILLIAMS, of Illinois, enclosing a preamble and resolutions, which he desired to read to the meeting. He then read the letter, of which the following is a copy:

*American Medical Biography, for the American Medical Association assembled at St. Louis.*

Mr. PRESIDENT: At the last meeting of the Association at New York, I presented the following preamble and resolution through my friend Dr. STEWART of New York, which, with some amendments were laid on the table:

As we are constantly called upon to deplore the ravages of death among the illustrious and worthy members of our profession throughout the United States.

*Resolved*, That a Standing Committee be appointed by the Association to procure memorials of the eminent and worthy dead among the distinguished physicians of our country, and present them to this Association for publication in the transactions.

I now beg leave to call up the resolution through my friend Dr.

J. B. JOHNSON, of St. Louis. The medical biography of our country is intimately related to the history of it, as the lives of eminent men are identified with the history of the times in which they lived. In the United States there have been, and are to be found, medical men whose lives and actions are ornaments to human nature, and whose brilliant career in the cause of humanity and science reflect honor and dignity upon our country. We suffer nothing in this respect in comparison with the learned and eminent physicians in Europe.

The profession of medicine contains more learned and distinguished men than any other profession or calling, and some memorial of their lives and actions should be persented to the world in a more durable form than the periodicals of the day, and particularly of the newspaper press. A more permanent and proper place for the publication of such memorials, would be in the Transactions of the American Medical Association, and without disparangement to any other articles which have heretofore been published in these Transactions—which memorials would be read by the members of the profession with great interest and improvement. Short biographies need take up but little room in the publication, and this objection to the proposed movement may be obviated. We are constantly noticing that death spares no rank or condition of men. Those who contend most skillfully against his insatiate ravages, themselves fall victims to his all conquering sword. Within a very short space of time, we have been called to lament the death of Dr. NATHAN CHAPMAN, the former President of this Association, of Dr. SAMUEL G. MORTON, WM. E. HORNER, ISAAC PARRISH, G. S. PATTISON, J. KEARNEY RODGERS, DAN. DRAKE, the great medical pioneer of the West, SAMUEL MCCLELLAN, AMOS SWITCHELL, ABIEL PIERSON, G. C. SHATTUCK, ARCHIBALD WELCH, and very many others which time will not permit me to enumerate.

This is not the place to speak their eulogies. Some permanent notice of them and of many others who have recently died, should be published in the Transactions of this Association, where the useful improvements and discoveries of the living should be recorded, and the memories of the worthy dead should be preserved. I hope the resolution will prevail.

[Signed,] STEPHEN W. WILLIAMS,

LAVINA, Winnebago county, Illinois, late of DEERFIELD, Massachusetts.

The Preamble and Resolutions referred to in the foregoing, were then on motion adopted. The Chair then announced that he would appoint the Committee, contemplated by the resolution, hereafter.

Dr. MCILVAINE offered the following resolution:

*Resolved*, That in the opinion of this Association, the practice of Professors reading lectures to their classes, no matter with how much care selected from the musty records of antiquity, is a miserable apology for teaching, is *prima facie* evidence of their inaptness to instruct, and is inimicable to medical progress.

It was, on motion, laid on the table.

Dr. FRENCH submitted the following resolution, which was carried:

*Resolved*, That a committee be appointed to enquire what State or other Society, represented in this Association, are in fellowship with irregular practitioners.

Dr. BLACHFORD, of Troy, read a letter from Dr. A. D. SPORE, stating that he (Dr. SPORE) had been for some time investigating the subject of Hydrophobia, to ascertain what influence the weather had upon the disease, and in the letter he requested that communications on the subject might be sent to him by members of the Faculty who had opportunities of making observations. Dr. SPORE not being a delegate it was moved that Dr. BLACHFORD be appointed Chairman of a Committee for the investigation of this subject.

The following resolution was offered by Dr. DOWELL:

*Resolved*, That a committee be appointed to investigate the improvements of the instruments for Lithotomy by NATHAN R. SMITH, PAUL F. EVE, and Dr. McDOWELL.

The resolution was laid on the table.

The following was offered by Dr. S. M. SMITH, of Columbus, Ohio, which was carried:

*Resolved*, That a Standing Committee of —— be appointed by the Association on the subject of Insanity as it prevails in this country, including its *causatire* as hereditary transmission, educational influences, physical and moral, social and political institutions, &c. Its forms and complications, curability and means of cure and prevention.

The announcement was now made that at 4 o'clock, P. M., there would be fifteen omnibuses at the door of the Hall awaiting the members of the Association, together with numerous buggies and carriages, to conduct them to the residence of COL. O'FALLON, who had extended them a general invitation to an entertainment.

The meeting adjourned to 3 o'clock in the afternoon.

---

AFTERNOON SESSION.

The Association met as per adjournment.

Dr. SAMUEL P. WHITE, of the University of Buffalo, submitted the following resolution, which was carried:

*Resolved*, That the thanks of this Association be presented to Dr. J. KNIGHT, late President, for the very dignified, courteous and efficient manner in which he presided over its deliberations, and that he be respectfully requested to furnish the usual address for publication.

The Committee appointed by the American Medical Association to devise or consider some comprehensive plan for the more general, systematic and thorough investigation of subjects connected with medical science, made a report, to which was appended the following resolution:

*Resolved*, That the American Medical Association hereby recommends all Medical Societies to establish, in accordance with the plan detailed in the foregoing report, special committees for the selection, investigation, elaboration and publication of all subjects of interest connected with medical science.

The resolution carried, and the report and resloution was referred to the Committee on Publication.

Dr. ATLEE communicated to the meeting that he had received a letter from Dr. PARISH, Chairman of the Committee on Epidemics of New Jersey, stating that his report was yet unfinished, but would soon be ready for publication.

On motion, it was directed to be handed over to the Committee on Publication when finished.

Mr. DAVIS presented some specimens of milk to the Association, which he explained would, if used, prevent many of the diseases to which children are subject, arising from using putrid milk. He respectfully submitted the specimens to the consideration of the Association.

After a session of half an hour, the meeting adjourned till nine o'clock next morning.

---

### THIRD DAY—THURSDAY.

The Association convened at nine o'clock, A. M. Dr. POPE, President, in the Chair.

On motion, the regular order of business was suspended for the purpose of filling the vacancy in the Nominating Committee from Iowa.

On motion, Dr. McGINGAN was chosen to fill the vacancy.

The minutes of Wednesday's proceedings were read, and after a few unimportant amendments, were adopted.

Dr. MCPHEETERS stated to the Association that arrangements had been made with the different lines of travel from the city, to convey the members of the Association to their homes, free of charge; and that all the lines and companies had agreed to the arrangement, excepting the New York and Hudson River Railroad Company.

The Secretary now read a communication from the Chairman of the Nominating Committee, requesting that body to meet. Also, a communication tendering the hospitality of the city of Burlington, Iowa, to those members who return by the Upper Mississippi.

Dr. Atlee offered the following resolution, which carried:

*Resolved*, That it shall be the duty of the Publication Committee to append to each volume of the transactions hereafter published, a copy of the Constitution of the Association.

The following resolution, offered by Dr. Gross, was also carried and Dr. Gross was appointed by the Chair the committee designated:

*Resolved*, That a Committee of one be appointed by the Chair to inquire into the causes which obstruct the formation and establishment of our National Medical Literature, and to report the subject at the next annual meeting of this Association, or as soon thereafter as practicable.

The following communination from the Engineer and Superintendent of the Pacific Railroad, was read by the President:

Dr. Washington, Chairman of the Committee of Arrangements.

*Dear Sir:* I am authorized by the Directors of the Pacific Railroad to offer to your Committee the use of the road, at your convenience, in case you should desire to show to the members of the Medical Society that are expected to meet here, the country in the neighborhood of St. Louis, and go a few miles out on what we hope will in time be the road to the Pacific Ocean.

If you will give me one day's notice, I will send out a special train, at such an hour as may suit your arrangements.

Very respectfully,

THOS. S. O'SULLIVAN,

Eng. and Sup't.

On motion of Dr. Atlee, a vote of thanks was extended by the Association to the Directors of the Pacific Railroad.

The Chairman of the Committee of Assangements moved that the Association accept the invitation of the Company—with the information that 10 o'clock, A. M., on Friday would be a convenient hour for the excursion to be made, as this would allow opportunity for delegates, who wished to leave the city in the afternoon, to be in season for boats, trains, &c.

Dr. J. Berrien Lindsley offered the following resolution, which, on motion, was referred to the Committee on Medical Education, with instructions to report at the next Annual Meeting of the Association:

*Resolved*, That this Association earnestly recommend to the few Western schools which still retain the rule of making four years' practice equivalent to one term at College, the abrogation of said rule, as holding out a strong inducement and temptation to young men to enter upon the practice of medicine with little or no preparation.

Dr. PAUL F. EVE, of Nashville, Tenn., submitted a resolution, which, after amendment, was carried:

*Resolved*, That a Committee of three be appointed by the Chair to report at the rext meeting of the Association, the best means for preventing the introduction of disease by emigrants into our country.

The Chair appointed Drs. DICKSON, GRISCOM, and E. D. FENNER the Committee.

Dr. LINTON, of St. Louis, offered the following resolution which was also referred to the above named Committee.

*Resolved*, That in the opinion of this Association, Quarantine establishments afford no protection to States and cities against the invasion of epidemics such as cholera and yellow fever.

Dr. PENN offered the following resolution, which was carried:

*Resolved*, That the members of the Committee of Arrangements who are not members of the Medical Association, be invited to take seats in this Association, as members by invitation.

Dr. JAYNE arose and offered a resolution in relation to the memorial from the American Medical Society at Paris, to the American Medical Association, which was, at a previous meeting, referred to the Committee on Education.

*Resolved*, That the memorial from Drs. HAMMER and MURPHY be withdrawn from the Committee on Education.

Dr. JAYNE spoke at some length regarding the memorial referred to and the object of his resolution.

He stated that such a document implied the inefficiency of American Professors of Medicine to give students a complete and thorough scientific education, and cast unjust and shameful reflections upon the capacity of medical men in this country. He contended that such a document, being so disposed of, appearing in the published proceedings of the American Medical Association, to be read in all parts of the Union, would confer a lasting disgrace on the judgment and deliberations of that body, and on the whole Faculty in this country. He warmly supported the object of the resolution, and submitted it.

Dr. Atlee remarked in this connection, that he knew something of the motives of the young men who wrote it, and was convinced that it was dictated by the best intentions, and without any desire on the part of the authors, to underrate the members of the profession, or medical science in this country.

Dr. Elbert entered warmly into the discussion, and remarked that the Association should act upon the document as it appeared. That it spoke for itself, and that the Association had nothing to do with intentions outside of the language of the document.

Dr. McIlvain wished the objection to the document more clearly specified than he had yet heard it. He believed it to have been sent in good faith.

Dr. Edgar then made some statements about the loose and incompetent education received by some Physicians in this country in certain schools, before they were considered qualified and permitted to enter upon the practice of the profession. He instanced two young men who had commenced the study of medicine in his office, and whom after remaining six or eight months he candidly told that they would never make competent Physicians. The young men left, and in eighteen months or two years from that time they were regular practicing physicians, having passed through some school which he did not name. He contended that some protection was necessary. He said, moreover, that an effort was made some time ago in this State to establish a Board of Censors, under whose examination every student should be required to pass before entering upon the duties of the profession, but that it failed, the physicians arising *en masse* against it, with the single exception of Dr. Linton

After some more discussion the memorial was withdrawn from the Committee of Education and laid on the table.

A communication was read from Dr. Peebles, chairman of the Committee on Epidemics of Virginia and North Carolina, requesting to be excused from the committee. The request was granted.

The Secretary stated that a document had been received from Dr. Phelps, of New York, purporting to be an abstract of a paper explaining the relation between medicine and religion.

On motion the regular business was suspended to give place to the reading of the report of the Nominating Committee, of which the following is a copy:

REPORT OF THE COMMITTEE ON NOMINATIONS.

The Committee on Nominations, in fulfilling the duty imposed upon them, recommend the continuance of several of the special committees previously created, and the appointment of some new ones. They, therefore, submit the following list of Chairmen of special committees, with the subjects to them committed:

Dr. Worthington Hooker, of New Haven, Conn., on Epidemics of New England and New York.

Dr. John L. Atlee, of Lancaster, Penn'a., on Epidemics of New Jersey, Pennsylvania, Delaware, and Maryland.

Dr. D. J. Cain, of Charleston, South Carolina, on Epidemics of South Carolina, Florida, Georgia, and Alabama.

Dr. W. L. Sutton, of Georgetown, Ky., on Epidemics of Tennessee, and Kentucky.

Dr. Thomas Reyburn, of St. Louis, Mo., on Epidemics of Missouri, Illinois, Iowa, and Wisconsin.

Dr. George Mendenhall, of Cincinnati, Ohio, on Epidemics of Ohio, Indiana, and Michigan.

Dr. E. D. Fenner, of New Orleans, La., on Epidemics of Mississippi, Louisiana, Arkansas, and Texas.

Dr. James Jones, of New Orleans, La., on the mutual relations of yellow and bilious remittent fever.

Dr. D. F. Condie, of Philadelphia, Penn'a., on the causes of Tuberculous disease.

Dr. Jos. Leidy, of Philadelphia, Pa., on diseases of the Parastic Origin.

Dr. A. P. Merrill, of Memphis, Tenn., on the Physiological Peculiarities and Diseases of Negroes.

Dr. Jos. N. McDowell, of St. Louis, Mo., on Statistics of the operation for the removal of Stone in the Bladder.

Dr. F. P. Porcher, of Charleston, South Calolina, on the Toxicological and Medicinal properties of our Cryptogamic plants.

Dr. Dan'l Brainard, of Chicago, Illinois, on the Constitutional and Local Treatment of Corcinoma.

Dr. Geo. Engleman, of St. Louis, Mo., on the Influence of Geological Formations on the Character of Disease.

Dr. Henry Taylor, of Mount Clemmens, Mich., on Dysentery.

Dr. Horace Green, of New York, on the use and effect of applications of Nitrate of Silver to the Throat in Local or General disease.

Dr. P. C. Gooch, of Richmond, Va., on the administration of Anæsthetic agents, during Parturition.

Dr. Chas. Hooker, of New Haven, Conn., on the diet of the sick.

Dr. E. R. Dabney, of Clarksville, Tenn., on certain forms of Eruptive Fevers, prevalent in Middle Tennessee.

Dr. Sanford B. Hunt, of New York, on the Hygrometrical State of the Atmosphere in various localities, and its influence on health.

Dr. Frank H. Hamilton, of Buffalo, New York, on the frequency of deformities in fractures.

Dr. M. M. Pallen, of St. Louis, Mo., on diseases of the Prostrate Gland.

Dr. H. A. Johnson, of Chicago, Illinois, on the Excretions as an index to the Organic changes going on in the system.

Dr. Leroy H. Anderson, of Sumpterville, Ala., on Typhoid Fever and its complications as it prevails in Alabama.

Dr. W. H. Byford, of Evansville, Ia., on the Pathology and Treatment of Scrofula.

Dr. N. S. Davis, of Chicago, Illi., on the Nutritive qualities of Milk, and the influence produced thereon by pregnancy and menstruation in the human female, and by pregnancy in the cow, and also on the question whether there is not some mode by which the nutritive constituents of milk can be preserved in their purity and sweetness, and furnish d to the inhabitants of cities in such quantities as to supercede the present defective and often unwholesome method of supply.

Dr. E. B. Haskins, of Clarkesville Tenn., on the Microscopical Investigations of Malignant Tumors.

Dr. Geo. K. Grant, of Memphis, Tenn., on the Sulphate of Quiuia as a remedial agent in tne treatment of Fevers.

Dr. R. R. McIlvain, of Cincinnati, Ohio, on the study of Pathology at the bedside.

Dr. E. S. Cooper, of Peoria, Ill., on Orthopædic Surgery.

Dr. Andrew F. Jeter, of Palmyra, Mo., on the modus operandi of the envenomed secretions of healthy animals.

Dr. S. M. Smith, of Columbus, Ohio, on Insanity.

Dr. Rene La Roche, of Philadelphia, Penn'a., on the Jaundice of Yellow Fever in its Diagnostical and Prognostical revelations.

Dr. Chas. Chandler, of Rocheport, Mo., on malignant periodic fevers.

Dr. R. S. Chase, of Portland, Maine, on Typhoid fever in Maine.

Committee on Plans of Organization for State and County Societies.—A. B. Palmer, Michigan; R. R. McIlvain, Ohio; D. L. McGugin, Iowa; E. R. Peaslee, New Hampshire; Thomas Lipscomb, Tenn.

Committee on Medical Literature.—Robert J. Breckenridge, Kentucky; A. A. Gould, Mass ; D. L. McGugen, Iowa; J. B. Flint, Ky.. O. M. Langdon, Ohio.

Committee on Medical E lucation.—Wm. H. Anderson, Ala.; A. Lopez, Ala.; Andrew Murray, Mich.; A. Ramsey, Tenn.; R. D. Ross.

Committee on Prize Essays.—Rene La Roche, Pa ; Isaac Hays, Pa.; Alfred Stille, Pa.; J. B. Biddle, Pa.; Geo. W. Norris, Pa.; Joseph Carson, Pa.; Joseph Leidey, Pa.

Committee of Arrangements—Isaac Hays, Pa.; G. Emmerson, Pa.; Wilson Jewell, Pa.; Alfred Stille, Pa.; Francis West, Pa.; Wm. V. Keating, Pa.

Committee on Publication.—Pliny Earle, New York; D. Francis Condie, Pa.; E. S. Lemoine, Mo.; A. March, N. Y.; E. H. Davis, N. Y.; C. R. Gilman, N. Y.

A warm discussion arose on the proposition to place a majority of physicians of New York on the Publication Committee, the effect of which would be to transfer the printing of the Transactions from Philadelphia to that city. The discussion continued for two or three hours, when the Association took a recess.

---

### AFTERNOON SESSION.

The Association convened at three o'clock. Dr. Pope in the Chair.

A resolution was offered by Dr. Storer in relation to permanent members, which was unanimously carried.

The Treasurer said that he had an announcement to make for the benefit of the friends of the Association. He stated that in the morning he had taken a ten guilder piece, mistaking it for five dollars, and had in his possession a three dollar doubtful bill, which the Brokers refused, and that one of the members had taken a receipt for three dollars without paying the money. He advocated the rectifying of these mistakes.

A resolution was offered to the effect that W. S. Maus, M. D., of Pekin, Ill., be elected a permanent member—which was carried unanimously.

Dr. Atlee offered the following resolution, which was carried:

*Resolved*, That this Association earnestly recommend to their medical brethren in those States in which Societies do not exist, the immediate organization of State and County Medical Societies.

Dr. Ramsey offered a resolution, which, on motion of Dr. Coons, was laid on the table.

Dr. Breckenridge offered a resolution to the following effect:

*Resolved*, That the papers and documents of the Association shall hereafter be the exclusive property of the Association.

Which, as amended by Dr. Smith, was carried.

Dr. Phelps, of New York, again requested to read the document which he presented in the morning, and the reading of which was deferred to allow the Committee of Nominations to make their report.

Mr. PHELPS took the stand, and read the following abstract:

The document I hold in my hand purports to be an abstract of a paper which traces the connection existing between medicine and religion in its origin and progress, and might receive the following style, to wit:

Religion an Element in Medicine, or the duties and obligations of the profession.

It naturally divides itself into parts; the former is mainly pictorial, the latter suggestive and practical. The general scope and bearing of the subject may be embraced under the following heads:

The substance and general outline may be stated in the following propositions:

1. A recognition of the complex nature of man, his immortality, free-agency, natural religious propensities, and susceptibility of indefinite intellectual improvement.

2. That medicine, or the art of healing, as an art, must have been of very ancient origin, probably cöeval with, or shortly subsequent to the fall of man; but, as a science, of much later date.

3. That the earliest authentic record of medicine and the profession, and the collateral art of the apothecary, is to be found in the Bible, and localized in Egypt, the great depository of learning and art in that early age.

4. That among the Israelites under the Theocracy, and down to the time of the Apostles, medicine and the profession were more or less identified with, or under the direction of the patriarchs, prophets, priests and evangelists, or other prominent and good men.

5. That the Greeks, though idolaters, were nevertheless deeply imbued with a sense of the claims and sacred obligations of their religious rights, and that Hippocrates and his disciples, as evidenced in the celebrated oath bearing his name, avouched the highest religious sanctions in entering upon the responsible duties of the profession of medicine.

6. That through the long night of the dark ages, the convulsions the vice and downfall of nations, science, medicine and religion feel the shock—languish and struggle for an uncertain existence, but are not destroyed; and with the dawning of the reformation and the consequent revival of learning, and the spirit of free inquiry, the arts, science and medicine as well as religion, receiving new impulses extend their domains, freedom of thought develops new elements of improvement, and the commonwealth of letters measurably disenthralled, gives lustre and momentum to knowledge, and high promise of success to succeeding generations.

7. That the art of printing—the discovery of a new continent—

of the circulation of the blood, and the publishing to the world the great principles of the Bactonian philosophy, together with the more general diffusion of the doctrines of the Reformation, gave new and increased energy and extension to thought, science, medicine, and the arts of civilized life, during the period included between the last half of the fifteenth and near the close of the seventeenth century.

8. That still another and brighter era dawned upon the world in the last half of the eighteenth century, in ascertaining the identity of the electric fluid and lightning—the discovery of galvanism of carbonic acid gas, of oxygen gas, and the consequent development of the broad field of pneumatic chemistry, and *pari passu* with these, the increased diffusion of Christian principles and benevolence as exhibited in the Bible, and other kindred societies—hospitals, almshouses, asylums and other numerous charitable institutions—very much of the efficiency of which depends upon the science, the time, the sacrifice and exposure of the medical profession.

9. That the scientific and other general developments of the last fifty years have been rapid above all human conception; every department and enterprise feels the mighty impulse, not an ocean or continent escapes the scrutiny of the age. The long standing problem proposed by Columbus, of a northwest passage, is solved. Air, steam, and electricity yield subserviency to the will of man and execute his pleasure. Time and space are measurably annihilated. Remote parts of the country are brought into neighborhood. Intercommunication, hitherto so difficult between distant nations, is facilitated; the benign principles of the Gospel wend their way; and hence the indication of the early universal diffusion of light and knowledge, and the final mental emancipation of our race.

10. That in elaborating the world's destiny, religion is the great and all pervading element, and that even now it exerts a controlling or modifying influence, commanding the homage and respect, either directly or indirectly, of all classes and professions.

11. That this influence is felt and receives the sanction and acknowledgment not only of the profession, as evidenced in our code of ethics, but specially also as witnessed in lectures, professors and presidents in their addresses, and other official acts in our colleges and schools of medicine.

12. That this moral influence as a necessary sequence in professional intercourse with the people and with each other, is exerted on man, complex in his nature, consisting of soul and body, and these reciprocal in their action and influence, and contingent in results here and hereafter, more or less upon professional intervention in the treatment of disease.

13. That hence we deduce inferentially, the solemn and responsible professional relation existing between the physician and his patients, and that in consonance both with the requirements of our code of ethics and the judgment of those high and honored in the profession, it is obligatory on the practitioner in grave disease, seasonably to take the initiatory demanded either for business arrangements, or leading to that consolation which religion can afford.

14. That the prudent and discreet inculcation of the truths of the Gospel and the method of man's redemption by a Mediator, instead of exerting an injurious effect upon bodily disease has often quite a contrary tendency, arresting the attention of the patient—leading to a different train of thought, and thus sensibly relieving anguish of mind and pain of body—begetting trust, confidence and joy and often ending in fatal cases in a peaceful and triumphant death.

15. That from the general scope of the subject, the compound nature of man, his eternity, accountability, religious propensities, intellectual susceptibility and the reciprocal action of body and mind in health and disease, the doctrine is educed of the paramount duty of the profession to their patients not only as regards the body in disease, but also the higher interests of the immortal spirit. And hence, also, the just claim of religion, the great anæsthetic of the immortal mind, to be considered an element in medicine or the healing art.

The foregoing propositions are enforced by such considerations and reflections as are suggested in support of the general subject.

New York. JAMES L. PHELPS.

The document was referred to a Special Committee, to be appointed by the Chair.

The President named Drs. Atlee, Sayre and March, to serve on the committee.

A motion was now made to proceed to the regular business, which was carried.

Dr. Eve moved that the matter relating to the report of the Committee on Nomination, and the blank occasioned by the amendment offered by Dr. Reyburn, which was adopted in Committee of the Whole, be referred back to the Nominating Committee for the purpose of filling up the blank, which was lost.

The question then came up upon adopting the original report of the Committee of Nomination. A discussion arose, participated in by Drs. Breckenridge, Atlee, Sayre Storer, McDowell, White, and others. It was in character much the same as that of the morning, becoming very animated, and at times personal feeling and sectional jealousy were evinced.

The original report of the Committee was at length adopted.

After the vote was announced the delegation from Philadelphia, through Dr. La Roche, announced that they would take the responsibility of tendering the resignation of Dr. Condie, of Philadelphia, Treasurer of the Association. After some little discussion the resignation of Dr. Condie was accepted.

Dr. West, of Philadelphia, one of the Secretaries, then tendered his resignation, and the question being upon accepting it it was lost.

Dr. Breckinridge, of Kentucky, then offered the following resolution, which was carried:

*Resolved*, That hereafter the majority of the Committee on Publication shall be selected from the physicians of that city in which the Association may annually meet.

A vote of thanks was then unanimously returned to Dr. Condie for the able, zealous and impartial manner with which he had discharged his duties as Treasurer.

A resolution was reported to amend the Constitution, which provides that its annual meetings shall be held on the first Monday in May, and substitute the second Monday. The resolution, under the rule, lies over for a year.

Dr. W. H. Byford offered a resolution of thanks to Dr. Pinckney, of the United States Navy, for the able address delivered before the Association on the first day of its session, which was unanimously adopted.

Dr. Atlee offered a resolution tendering the thanks of the Association, and of the individual members, to the citizens of St. Louis for their hospitality and kindness; also, to the Directors of the various Railroads and officers of steamboats, for the generous manner in which they have tendered their kind offices, which was adopted.

Dr. White moved that a vote of thanks be extended to the late Publishing Committee for their best endeavors to serve the Association, which was unanimously adopted.

A resolution offered by Dr. W. W. Hitt, about alcoholic drinks, was adopted and referred to the Committee on Nomination.

A resolution offered by Dr. C. B. Hughes, relating to speciality practice in Surgery, was on motion laid on the table.

Dr. White, from the Nominating Committee, reported to the Association the name of Dr. Blatchford, of New York, as Treasurer, in place of Dr. Condie, resigned.

Dr. Blatchford declined acting in that capacity, and the Committee subsequently reported the name of Dr. Wood, of New York.

They also reported a special committee on Epidemics for the States of Virginia and North Carolina, with Dr. Haskins as chairman; and also, the resolution relative to alcoholic drinks was reported back by them, referring it to a special committee, consisting of Dr. Mussey.

Dr. W. S. Edgar offered a resolution in regard to the compounding of medicine, and recommending apothecaries to use different colored paper in putting up poisonous drugs, with an appropriate stamp upon it, in contradistinction to other medicines.

Dr. Bane, of Illinois, was elected a permanent member.

A letter from Dr. Engelman, of St. Louis, was read, resigning his situation as chairman on one of the special committees, but the Association refused to accept it.

The following resolution, offered by Dr. J. B. Lindsley, was, on motion, laid on the table:

*Resolved*, That the too prevalent practice of Professors in Medical Colleges of recommending their own writings and editings as text books for their students, is, in the opinion of this Association a serious evil, trammeling as it does, the student in his choice of books, and promoting the publication and circulation of works of inferior merit.

A vote of thanks was returned to Dr. Hooker, Treasurer, *pro. tem.*

Dr. Gross informed the Association that the second volume of the work of the late Prof. Drake, of Cincinnati, was now in the press at Philadelphia, and would be issued early in the present summer. The second volume he said was on Practical Medicine, and will be entirely independent of the first.

Dr. McPheeters announced to the Association that omnibuses would be in attendance at the Hall next morning at nine o'clock, to convey the members to the depot of the Pacific Railroad, in pursuance of the invitation tendered to them by the Directors.

On motion the Association then adjourned *sine die.*

In the evening the members sat down to a magnificent entertainment provided by the physicians of St. Louis, which "passed off in a very orderly and satisfactory manner," with toasts, speeches, poems, stories, and songs, affording to all present high gratification.

